# The Association between Cerebral Oxygenation and CKD in Older Adults

**DOI:** 10.34067/KID.0000001117

**Published:** 2026-01-15

**Authors:** Caoimhe McGarvey, Robert Briggs, Louise Newman, Siobhan Scarlett, Aisling M O'Halloran, Cathal McCrory, Rose Anne Kenny, Donal J. Sexton

**Affiliations:** 1 The Irish Longitudinal Study on Ageing (TILDA), School of Medicine, Trinity College Dublin, Dublin, Ireland; 2 Mercer's Institute for Successful Ageing (MISA), St James's Hospital, Dublin, Ireland; 3 School of Nursing and Midwifery, Trinity College Dublin, Dublin, Ireland; 4 Renal Unit, St James's Hospital, Dublin, Ireland

**Keywords:** CKD, chronic renal disease, cognition, complications, dementia

## Abstract

**Key Points:**

- Older adults with CKD had significantly and independently lower dynamic cerebral oxygenation at all time points on standing.
- When analyzed by eGFR level, lower eGFR levels were consistently associated with reduced dynamic cerebral oxygenation.
- Lower cerebral oxygenation in CKD may drive cognitive decline and serve as a marker for risk stratification.

**Background:**

CKD is common, and its prevalence is increasing rapidly worldwide. The aim of this study was to investigate the relationship between CKD and dynamic changes in cerebral oxygenation, which may help explain the link between CKD, cerebrovascular disease, and cognitive impairment.

**Methods:**

This observational study used data from waves three (2014–2015) and six (2020–2023) of the Irish Longitudinal Study on Aging, a nationally representative, population-based cohort of community-dwelling adults aged 50 years or older in Ireland. A total of 2322 participants (mean age 64.7±7.6 years; 53% female) were included. 7.1% had CKD, defined by eGFR. Cerebral oxygenation, indicated by the tissue saturation index, was continuously measured using near-infrared spectroscopy during and after an orthostatic maneuver.

**Results:**

Participants with CKD were older, had lower levels of educational attainment, and higher prevalence of cardiovascular and cerebrovascular disease and cognitive impairment. At wave three, cerebral oxygenation was significantly and independently lower at all time points post-standing in those with CKD (30 s:−0.23 [95% confidence interval, −0.43 to −0.04], 60 s:−0.31 [−0.51 to −0.11], 90 s −0.34 [−0.54 to −0.14], 120 s: −0.34 [−0.54 to −0.14], 150 s: −0.32 [−0.52 to −0.12], and 180 s: −0.36 [−0.55 to −0.16], *P* < 0.05). The coexistence of orthostatic hypotension amplified this association at later time points, while systolic hypotension may mitigate it. Lower eGFR was associated with lower cerebral oxygenation; eGFR 45–59 ml/min per 1.73 m^2^ showed the most pronounced decline.

**Conclusions:**

CKD is significantly and independently associated with reduced cerebral oxygenation during orthostatic challenge. This may represent a key mechanism linking CKD to cognitive impairment. Further longitudinal studies are warranted to explore the clinical utility of cerebral oxygenation as a predictive biomarker for cognitive decline in CKD.

## Visual Abstract

**Figure:**
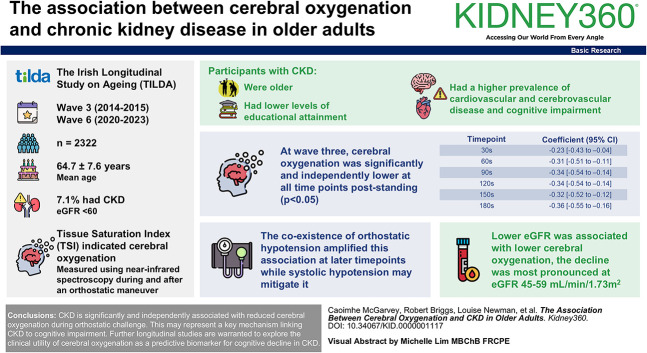

## Introduction

CKD is common and is increasing in prevalence with the rapidly aging global population.^1^ Kidney disease is strongly associated with aging, yet despite its increasing prevalence and significant health implications, it remains under-recognized by both the public and the health care community.^2^ Approximately 700 million people worldwide have CKD with a further 150 million having AKI or kidney failure.^3^ The World Health Organisation anticipates that CKD will be the fifth most common chronic disease globally by 2040.^4^ Concerningly, deaths due to kidney disease increased by 95% between 2000 and 2021 with CKD now being the seventh most common cause of death from noncommunicable disease worldwide.^5,6^ In Ireland, one in seven people older than 50 years have CKD, and most are unaware they have it.^7^

It is well established that CKD is associated with a higher risk of developing cognitive impairment.^8,9^ It has been postulated that this association may be due to the vascular mechanisms, as well as the impaired clearance of uremic metabolites, at play in CKD.^9^ People with CKD have higher rates of cerebrovascular disease, stroke, cognitive impairment, and dementia than the general population.^10^ The kidneys and brain are uniquely and similarly susceptible to vascular injury because of their high blood flow demand and reliance on autoregulation.^11^ Cerebral hemodynamic instability with disturbed blood flow regulation likely contributes to cognitive impairment in CKD.^12^ This study aims to investigate the relationship between CKD and dynamic responses in cerebral oxygenation, measured by Near-Infrared Spectroscopy (NIRS). This relationship may elucidate the known association between cerebrovascular disease, cognitive impairment, and kidney disease.

## Methods

### Study Design

This is an observational study that examines the association between CKD and cerebral oxygenation using data from the Irish Longitudinal Study on Aging (TILDA). TILDA is a population-based prospective cohort study of a nationally representative sample of community-dwelling adults aged 50 years and older, living in Ireland. The initial study population involved more than 8000 participants. The TILDA study design has been described previously.^13^ Ethical approval for the study was received from the Trinity College Research Ethics Committee, and all participants provided written informed consent. All experimental procedures adhered to the Declaration of Helsinki, and health assessments were performed by trained health care professionals.

This study analyzed data from waves three and six of TILDA which were collected between 2014–2015 and 2020–2023, respectively. Data analysis for this study took place from September 2024 to August 2025. All participants older than 50 years, who had a health assessment with complete measurements of orthostatic BP, cerebral oxygenation measured by NIRS and markers of kidney function, specifically, creatinine and cystatin C were included in the final analyses at each wave. The wave six follow-up analysis has fewer participants because participants were randomized to undergo either health assessment with cerebral oxygenation or detailed cognitive assessment at this wave.

### Cerebral Oxygenation and Orthostatic BP

As part of the health assessment, each participant underwent an active stand test, this took place in a quiet room that was kept at an ambient temperature. Participants lay in a supine position for 10 minutes and were then asked to stand promptly, with assistance provided if needed. Participants remained standing for 3 minutes. Both cerebral oxygenation and beat-to-beat BP were measured simultaneously during the active stand at both waves three and six.

Cerebral oxygenation was measured continuously from the frontal lobe using the Artinis Portalite System, a portable, noninvasive NIRS system. It was first reported by Jobsis in 1977 that the transparency of brain tissue in the near-infrared spectrum allows for the measurement of tissue oxygenation using transillumination spectroscopy.^14^ The Portalite system uses three transmitters and one receiver, each transmitter emits two different wavelengths of light that are subsequently absorbed at different rates by oxygenated and deoxygenated hemoglobin, and their concentrations can be calculated on the basis of the principle of the absorption of light as described by the modified Beer-Lambert law.^15^

A single sensor was placed on the forehead of the participant at the FP1 position in the international 10–20 electrode system and measured frontal lobe oxygenated and deoxygenated hemoglobin continuously. The sensor was covered by a headband to avoid interference from environmental light. Tissue saturation index (TSI) was calculated as the oxygenated hemoglobin value expressed as a percentage of the total oxygenated and deoxygenated hemoglobin values and was the principal measure of cerebral oxygenation used in this study. Continuous beat-to-beat BP was measured by a Finometer (Finapres Medical Systems, Arnhem, Netherlands), which has been described previously.^16^ Signal processing, including an 11-point median filter and ten-point moving average filter, was applied to both BP and NIRS data to allow for accurate comparison. The sampling rate of the output data were 1 Hz.

Orthostatic hypotension (OH) was defined as a drop in systolic BP ≥20 mm Hg or drop in diastolic BP ≥10 mm Hg at 30, 60, or 90 seconds after standing compared with baseline BP.^17^ Baseline values for TSI and BP were calculated from the mean values of the readings during the last 30–60 seconds of the supine rest period. TSI was measured continuously during the active stand up to 180 seconds.

### Kidney Function

A venous blood sample was taken from consenting participants. Cystatin C and creatinine were measured simultaneously from frozen plasma using particle-enhanced and enzymatic immunoturbidimetric assays as described previously.^18^ eGFR was calculated with the 2021 CKD-Epidemiology Collaboration equation using a combination of the creatinine and cystatin C measurements at wave three only. CKD was defined as CKD-Epidemiology Collaboration eGFR <60 ml/min. For the purposes of analysis, eGFR levels were based on the Kidney Disease Improving Global Outcomes (KDIGO) classification system. Level 1 corresponds to KDIGO group 1, eGFR ≥90 ml/min per 1.73 m^2^; level 2 corresponds to KDIGO group 2, eGFR 60–89 ml/min per 1.73 m^2^; level 3 corresponds to KDIGO group 3a, eGFR 45–59 ml/min per 1.73 m^2^; and level 4 is a combination of KDIGO levels 3b, 4, and 5 or eGFR <45 ml/min per 1.73 m^2^ because of small participant numbers in these levels. Urinary protein excretion was not available in this study.

### Other Covariates

Demographic covariates included age, sex, and highest educational attainment. Baseline cardiovascular risk factors and history, including current smoking status, problematic alcohol consumption, diabetes status, stroke, and transient ischemic attack (TIA) history, and established cardiac diseases, including myocardial infarction, angina, and cardiac failure, were recorded. Problematic alcohol consumption was defined as a score of >2 on the Cutdown, Annoyed, Guilt, Eye-Opener questionnaire.^19^ The presence of diabetes, history of cardiac disease, TIA, and stroke were based on the self-report of a doctor’s diagnosis of the condition. Chronic disease number was based on the self-report of a doctor’s diagnosis of the following conditions: diabetes, arthritis, cancer, liver disease, lung disease, and kidney disease.

Antihypertensive medication use was recorded during the computer assisted personal interview and was coded according to the World Health Organization anatomical therapeutic chemical classification system. Antihypertensive medication was defined as medications categorized as C02, C03, C07, C08, and C09 using the anatomical therapeutic chemical classification system regardless of their treatment indication. Mini-Mental State Examination (MMSE) and Montreal Cognitive Assessment (MOCA) scores were also recorded, and cognitive impairment was defined as an MMSE score of <25 or a MOCA score of <26.^20^

Depression was assessed in the computer assisted personal interview using the eight-item Center for Epidemiological Studies Depression Scale (CES-D-8).^21^ This shorter version of the CES-D scale was used in the TILDA study to reduce the time taken to conduct participant assessments; previous work confirmed its reliability in comparison with the 20-item CES-D within the TILDA cohort.^22,23^ Depression was defined as a score of nine or more on the CES-D-8. Height and weight were measured by the study health practitioner, and body mass index was calculated as weight in kg/height in m^2^. Timed-Up-and-Go was measured during the health assessment, participants stood up, walked 3 m at normal pace to a line on the floor, turned around, walked back to the chair, and sat down. This was timed using a stopwatch.

BP measurements were taken according to a standard protocol at an ambient temperature of 20–25°C. A digital automated oscillometric BP monitor (Omron M10-IT, Omron Inc., Kyoto, Japan) with an arm cuff (22–42 cm) was used to measure BP in one arm, at heart height, while the respondent was seated comfortably in an upright position after a period of rest. BP was recorded twice while seated with a timed interval of 1 minute between readings. The mean systolic BP (SBP) was obtained from these two measurements. Hypertension was defined as a mean SBP value ≥140 mm Hg and/or mean diastolic BP value ≥90 mm Hg. Transition time or standing speed was calculated on the basis of changes in the height correction data during the active stand which enables the identification on when the stand starts and ends as described previously.^24^

### Follow-up Analysis

Six hundred thirty-six of the wave three participants had follow-up TSI measurements available at wave six (mean follow-up 6.5 (±0.4) years). Only 25 participants (3.9%) in the follow-up cohort had CKD; therefore, the follow-up analysis was performed for eGFR level only. Kidney function markers were not measured at wave six; therefore, the follow-up analysis is based on TSI measurements only.

### Statistical Analysis

Data analysis was conducted using Stata 15.1. Participant characteristics were described as mean and 95% confidence interval and compared by the Student *t* test in the case of continuous variables. For categorical variables, characteristics were presented as number and percentage of participants and compared using the chi-squared test.

Multilevel models with TSI as the dependent variable were used to compare TSI data across specific 10-second time points after standing by both the presence or absence of CKD and by eGFR level. These models assess the effect of the two-way interaction between CKD status or eGFR level and time (CKD status/eGFR level×time) on TSI. This accounts for the fact that TSI values at different time points are correlated with each other. The same process was followed to compare drop in TSI at various time points using both wave three and wave six data.

Models were adjusted for numerous covariates which were chosen on the basis of previous research that identified them as having significant associations with cerebral oxygenation, measured by NIRS. The first model was unadjusted, and the second model was adjusted for age, sex, height, and weight. The third model was adjusted for age, sex, education, weight, height, cardiac disease, stroke history, cognitive impairment, depression, hypertension, taking antihypertensive or antidepressant medications, diabetes, smoking history, alcohol excess, Timed-Up-and-Go, and standing speed. In the wave three CKD analysis, model 3 was further adjusted for OH and low systolic BP to assess the role of BP in mediating the relationship between TSI and CKD. This involved the addition of OH and SBP separately to the models and assessing the subsequent three-way interactions. A *P* value ≤ 0.05 was considered statistically significant in this study.

## Results

A total of 2322 participants were eligible for inclusion in this study. Overall, the mean age was 65 (±8) years, and 53% of participants were female. Seven percent of participants had CKD as defined by eGFR. Participants with CKD were older, had lower levels of educational attainment, and were more likely to have cardiac disease, as well as a higher number of chronic diseases, including diabetes. They were less likely to be current smokers. There was no significant difference between the two groups regarding the prevalence of depression or alcohol abuse. Regarding cerebrovascular disease and cognitive impairment, participants with CKD were more likely to have a history of stroke and TIA and were more likely to have cognitive impairment identified on MOCA or MMSE.

From a BP point of view, participants with CKD were more likely to be taking antihypertensive medications and had higher rates of OH at all time points during the active stand. Although those with CKD tended to have a higher mean SBP, this was not statistically significant, likely because of smaller numbers in the CKD group. Body mass index was significantly higher in the CKD group. Participant characteristics by CKD status are displayed in Table 1.

**Table 1:** Participant characteristics by CKD status (defined as 2021 CKD-Epidemiology Collaboration eGFR creatinine and cystatin C combination >60 ml/min per 1.73 m^2^)

Patient Characteristic	No CKD (*n*=2158)	CKD (*n*=164)
Age, yr, mean (95% CI)	64 (64 to 65)	72 (71 to 73)
Female, % (*n*)	53 (1141)	55 (90)
**Education, % (*n*)**		
Primary/none	15 (312)	31 (51)
Secondary	41 (881)	34 (55)
Tertiary	45 (965)	36 (59)
**Cardiac disease, % (*n*)**	6 (119)	21 (34)
Myocardial infarct	3 (72)	13 (21)
Cardiac failure	0.4 (9)	2 (4)
Angina	3 (63)	9 (15)
**Chronic disease number, % (*n*)**		
0	62 (1346)	45 (73)
1	33 (709)	44 (72)
2	5 (100)	10 (16)
3	0.1 (3)	2 (3)
Diabetes, % (*n*)	5 (116)	15 (24)
**Smoking history, % (*n*)**		
Never	49 (1053)	42 (69)
Ex-smoker	42 (898)	52 (89)
Current	9.6 (207)	4 (6)
Alcohol abuse, % (*n*)	14.5 (313)	7 (12)
Stroke history, % (*n*)	1.1 (23)	4 (6)
TIA history, % (*n*)	2.6 (57)	7 (12)
MMSE, points, mean (95% CI)	29.1 (29.0 to 29.1)	28.7 (28.4 to 28.9)
Cognitive impairment, % (*n*)	33 (707)	52 (86)
Depression, % (*n*)	7 (156)	9 (14)
Antihypertensive use, % (*n*)	34 (723)	72.0 (118)
BMI, kg/m^2^, mean (95% CI)	28.1 (27.9 to 28.3)	30.2 (29.4 to 31.0)
SBP, mm Hg, mean (95% CI)	133 (132 to 133)	135 (131 to 138)
**Orthostatic hypotension, % (*n*)**		
30 s, % (*n*)	12 (263)	24 (39)
30 and 60 s, % (*n*)	6 (133)	17 (27)
30, 60, and 90 s, % (*n*)	4 (88)	13 (22)

BMI, body mass index; CI, confidence interval; MMSE, Mini-Mental State Examination; SBP, systolic BP; TIA, transient ischemic attack.

**Figure 1:**
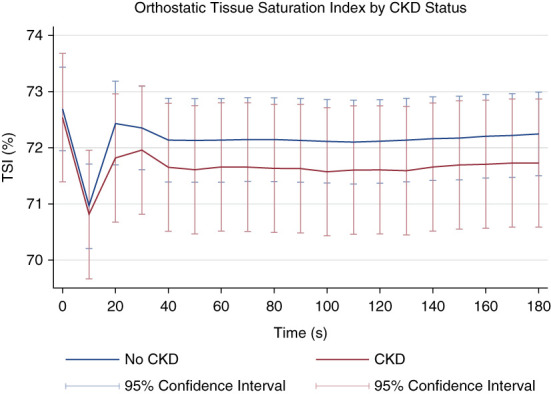
**Orthostatic TSI stratified by CKD status.** Figure 1 shows TSI, the measurement of cerebral oxygenation used in this study, on the *y*-axis with time after standing on the *x*-axis. Participants with CKD are represented by the red line, while those without CKD are represented by the blue line. The lines represent the predicted TSI values derived from multilevel modeling with TSI as the dependent variable nested within the participant. 95% CI are represented by capped lines. Models were fully adjusted as per model 3 in Table 2. TSI was measured by NIRS, and CKD status was based on a 2021 CKD-EPI eGFR of <60 ml/min per 1.73 m^2^ using a combination of creatinine and cystatin C measurements. CI, confidence interval; CKD-EPI, CKD-Epidemiology Collaboration; NIRS, near-infrared spectroscopy; TSI, tissue saturation index.

**Figure 2:**
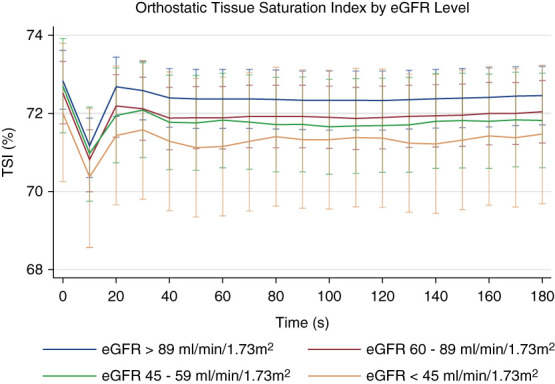
**Orthostatic TSI stratified by eGFR Level.** Figure 2 shows the TSI on the *y*-axis with time after standing on the *x*-axis. Participants are stratified by eGFR which was measured using the 2021 CKD-EPI formula using a combination of creatinine and cystatin C measurements. The blue line represents those with eGFR >89 ml/min per 1.73 m^2^, the red line represents those with eGFR 60–89 ml/min per 1.73 m^2^, the green line represents those with eGFR 45–59 ml/min per 1.73 m^2^, and the yellow line represents those with eGFR <45 ml/min per 1.73 m^2^. The lines represent the predicted values from multilevel modeling with TSI as the dependent variable nested within the participant. 95% CI are represented by capped lines. Models were fully adjusted as per model 3 in Table 2. TSI was measured by NIRS.

Figure 1 shows the pattern of change in TSI at specific time points after standing by CKD status, while Supplemental Figure 1 demonstrates the pattern of change in TSI drop from baseline at specific time points after standing by CKD status, as derived from fully adjusted multilevel mixed effects models. Figure 2 and Supplemental Figure 2 display the changes after standing in mean TSI and TSI drop, respectively, by eGFR level. The mixed-effects regression models for CKD status with TSI as the dependent variable demonstrated that TSI after standing was significantly lower in those with CKD at all timepoints. The significance of this association was maintained after adjustment for demographic and clinical covariates as detailed in Table 2 (models 2 and 3).

**Table 2:** Mixed-effects linear regression models for CKD status with tissue saturation index at wave three as the dependent variable

TSI	Coefficient (95% CI)	SE	*Z* Score	*P* Value
**Model 1—CKD status×time**				
30 s	−0.23 (−0.43 to −0.04)	0.10	−2.31	0.02
60 s	−0.31 (−0.51 to −0.11)	0.10	−3.06	0.002
90 s	−0.34 (−0.54 to −0.14)	0.10	−3.37	0.001
120 s	−0.34 (−0.54 to −0.14)	0.10	−3.33	0.001
150 s	−0.32 (−0.51 to −0.12)	0.10	−3.13	0.002
180 s	−0.36 (−0.55 to −0.16)	0.10	−3.50	<0.001
**Model 2—CKD status×time**				
30 s	−0.23 (−0.43 to −0.04)	0.10	−2.31	0.02
60 s	−0.31 (−0.51 to −0.11)	0.10	−3.06	0.002
90 s	−0.34 (−0.54 to −0.14)	0.10	−3.37	0.001
120 s	−0.34 (−0.54 to −0.14)	0.10	−3.33	0.001
150 s	−0.32 (−0.52 to −0.12)	0.10	−3.13	0.002
180 s	−0.36 (−0.55 to −0.16)	0.10	−3.50	<0.001
**Model 3—CKD status×time**				
30 s	−0.23 (−0.43 to −0.04)	0.10	−2.31	0.02
60 s	−0.31 (−0.51 to −0.11)	0.10	−3.06	0.002
90 s	−0.34 (−0.54 to −0.14)	0.10	−3.37	0.001
120 s	−0.34 (−0.54 to −0.14)	0.10	−3.33	0.001
150 s	−0.32 (−0.52 to −0.12)	0.10	−3.13	0.002
180 s	−0.36 (−0.55 to −0.16)	0.10	−3.50	<0.001
**Random-effects parameters**	**Estimate (95% CI)**	**SE**		
Variance of constant	23.2 (21.9 to 24.6)	0.68		
Variance of residual	0.78 (0.77 to 0.79)	0.01		
**Model 4: OH added to model 3 CKD status×time×OH**				
30 s	−0.40 (−0.87 to 0.08)	0.24	−1.65	0.10
60 s	−0.21 (−0.67 to 0.27)	0.24	−0.86	0.39
90 s	−0.23 (−0.71 to 0.25)	0.24	−0.95	0.34
120 s	−0.33 (−0.81 to 0.14)	0.24	−1.37	0.17
150 s	−0.52 (−0.99 to −0.04)	0.24	−2.13	0.03
180 s	−0.58 (−1.06 to −1.04)	0.24	−2.39	0.01
**Model 5: SBP added to model 3 CKD status×time×SBP <120**				
30 s	0.13 (−0.33 to 0.59)	0.24	0.54	0.59
60 s	0.38 (−0.09 to 0.84)	0.24	1.60	0.11
90 s	0.43 (−0.04 to 0.89)	0.24	1.81	0.07
120 s	0.33 (−0.13 to 0.80)	0.24	1.42	0.16
150 s	0.30 (−0.16 to 0.76)	0.24	1.26	0.21
180 s	0.43 (−0.04 to 0.89)	0.24	1.80	0.07

Model 1: Unadjusted.

Model 2: Adjusted for age, sex, weight, and height.

Model 3: Adjusted for age, sex, weight, height, education, diabetes, hypertension, cardiac disease, taking antihypertensive or anti-depressant medication, depression, stroke history, cognitive impairment, smoking and alcohol history, standing speed, and timed-up-and-go.

Model 4: Adjusted as per model 3 with interaction for orthostatic hypotension.

Model 5: Adjusted as per model 3 with interaction for low systolic BP

CI, confidence interval; OH, orthostatic hypotension; SBP, systolic BP; TSI, tissue saturation index.

Model 3 was additionally adjusted for the presence of OH or low SBP, and significance was maintained. This relationship was then explored in more detail with the addition of OH and low SBP separately to the interaction terms in models 4 and 5. The addition of OH in model 4 (Table 2), to the interaction between TSI, CKD, and time, resulted in the attenuation of the *z* scores at all time points, and this was statistically significant at 150 and 180 seconds. This finding suggests that the presence of OH affects the relationship between TSI, CKD and time, amplifying the decrease in TSI with time in CKD, and this is more pronounced at later time points in the active stand. The addition of low SBP to model 5 (Table 2), resulted in the transition of coefficients from negative to positive values as well as the attenuation of the *z* scores. Although these findings were not statistically significant at any time point, they suggest that the presence of low SBP may mitigate the effect of CKD on TSI over time.

**Figure 3:**
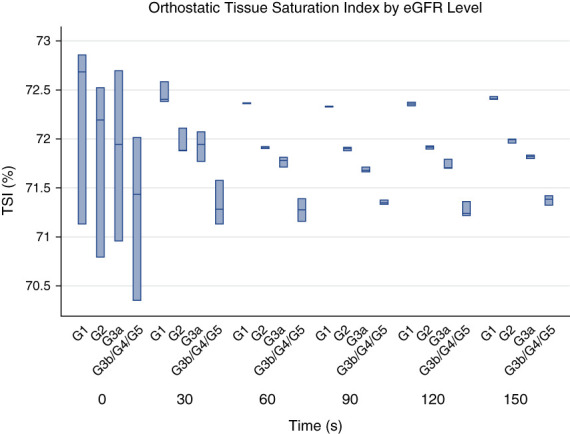
**Box plot of orthostatic TSI by eGFR level.** Figure 3 displays a box plot of the TSI expressed as a percentage on the *y*-axis with the time in seconds after standing on the *x*-axis, stratified by eGFR level. eGFR was measured using the 2021 CKD-EPI formula using a combination of creatinine and cystatin C measurements. G1 represents those with eGFR >89 ml/min per 1.73 m^2^, G2 represents those with eGFR 60–89 ml/min per 1.73 m^2^, G3a represents those with eGFR 45–59 ml/min per 1.73 m^2^, and G3b/G4/G5 represents those with eGFR <45 ml/min per 1.73 m^2^. The model output was fully adjusted as per model 3 in Table 2. TSI was measured by NIRS.

The fully adjusted model in Supplemental Table 1 shows that when analyzed by eGFR level, lower eGFR levels were consistently associated with reduced TSI after standing. This effect was strongest for level 3 eGFR while level 2 eGFR also showed a persistent and significant reduction in TSI. Although level 4 eGFR also demonstrated lower TSI values when compared with the reference group (level 1 eGFR), statistical significance was intermittent likely because of the smaller participant numbers in this group. This pattern is displayed in Figure 3.

## Discussion

This large population study has shown for the first time that there is a graded association between lower dynamic cerebral oxygenation and CKD, determined by eGFR level. Participants with CKD had significantly lower cerebral oxygenation after standing than the general population in a cohort of community-dwelling adults older than 50 years. This finding was persistent after multivariable adjustment as seen in Figure 1 and Table 2. Those with kidney disease experienced a greater drop in cerebral oxygenation after standing throughout the active stand, as displayed in Supplemental Figure 1. In multilevel mixed effects models, TSI was significantly lower in those with CKD at all time points. Further analyses examined the effect of BP patterns on these findings with the addition of OH and low SBP to the interaction term in models 4 and 5. This resulted in the attenuation of the *z* scores in both models; however, this only reached statistical significance in case of OH at 150 and 180 seconds.

When TSI, the marker of cerebral oxygenation used in this study, was compared by eGFR level, lower eGFR was associated with lower cerebral oxygenation, as shown in Figures 2 and 3. Detailed model output with coefficient and *P* values is displayed in Supplemental Table 1. On stratifying TSI drop by eGFR in Supplemental Figure 2, the pattern was not as clear; those with eGFR 45–60 ml/min per 1.73 m^2^ experienced a greater drop in TSI on standing compared with those with eGFR <45 ml/min per 1.73 m^2^. This may in part be explained by the compensatory mechanisms aimed at maintaining cerebral oxygenation in the setting of anemia in more advanced CKD.^25^ However, hemoglobin levels were not available in this study. Alternatively, this finding may be related to sample sizes in each eGFR category comparisons.

Anemia is common in people with CKD, approximately 15% of those with CKD have anemia. Prevalence increases from CKD Stage 4 onwards and from CKD Stage 3b in diabetes while most people with ESKD have anemia.^26,27^ Anemia reduces the blood's oxygen carrying capacity resulting in decreased oxygen delivery to tissues and organs, including the brain, thereby compromising cerebral oxygenation.^28^ This can result in a cascade of events including tissue hypoxia and oxidative stress with resultant compensatory mechanisms involving increased cerebral blood flow (CBF) and reduced blood viscosity.^28^ Although these compensatory mechanisms do exist as part of cerebral autoregulation, they are not always sufficient to maintain cerebral oxygenation and prolonged anemia can exhaust this compensatory capacity.^28^ It is likely that anemia would amplify the association between CKD and lower cerebral oxygenation found in this study and that this effect would be more pronounced in later CKD stages. With time, compensatory mechanisms may be reflected by increased cerebral oxygenation in ESKD but this effect may be transient.

The dynamic response to standing is highly relevant physiologically to cerebral oxygenation and CKD. We stand 30–50 times daily and have evolved sophisticated baroreflex systems to ensure preservation of cerebral and other organ blood flow during rapid postural changes. These mechanisms decline with age, exposing end organs to damage and dysfunction. Previous studies examining the association between CKD and cerebral perfusion have focused mainly on CBF measurements at rest, acquired by magnetic resonance imaging (MRI) and positron emission tomography–computed tomography.^25,29–33^ One previous study used transcranial Doppler to investigate the relationship between CKD and cerebral hemodynamics; however, this was based solely on a post-stroke cohort.^34^

On the basis of findings from the Rotterdam Study, Sedaghat *et al*. demonstrated that lower eGFR was independently associated with lower CBF when measured by MRI at rest in a study population of 2645 participants that excluded those with ESKD.^30^ In 2016, Tamura *et al*. found that in 75 participants with eGFR <45 ml/min per 1.73 m^2^, there was an association with a higher CBF measured by MRI at rest.^31^ Previous studies have demonstrated that CBF measured by MRI is initially higher in those with ESKD compared with controls but declines after 6 months of hemodialysis or peritoneal dialysis commencement.^32,35^ CBF was also shown to be increased in ESKD in a case-control study with 97 participants with ESKD, by Jiang *et al*. in 2016.^36^ This finding was negatively correlated with hemoglobin level.^36^ It has been hypothesized that the development of anemia in ESKD, which causes reduced blood viscosity and oxygen carrying capacity, induces compensatory mechanisms with increased CBF to restore cerebral oxygenation.^25^ It is also possible that more marked hypertension and increased mean arterial pressure in patients with ESKD could lead to increased blood flow, ultimately leading to maladaptation and cerebrovascular disease.

Studies using NIRS to measure cerebral oxygenation in those with CKD have demonstrated that lower eGFR and CKD are independently associated with lower cerebral oxygenation at rest and that cerebral oxygenation decreases significantly after the formation of arteriovenous fistula.^37,38^ However, these studies were limited to selected patient cohorts and included 68 and 48 participants, respectively. One previous study with 90 participants, by Theodorakopoulou *et al*. in 2023, examined cerebral oxygenation and regional blood volume changes using NIRS during a handgrip exercise and found that cerebral oxygenation increased less during this exercise with advancing CKD.^39^ To the authors' knowledge, this is the only previous study to examine a dynamic response in cerebral oxygenation, measured by NIRS, in people with CKD.

Abnormal CBF is known to be a precursor for cognitive impairment.^40^ The Rotterdam Study found that low CBF in the middle cerebral artery, measured by transcranial Doppler, is a risk factor for cognitive decline and atrophy.^41^ The association between CKD and cognitive impairment and the higher rates of cerebrovascular disease in patients with CKD are well-documented.^8–10^ Our study reproduces these findings and demonstrates lower dynamic cerebral oxygenation in response to standing in participants with CKD, highlighting the need for the consideration of reduced cerebral oxygenation and altered cerebral hemodynamics as a key mechanism underlying the increased rates of cognitive impairment and cerebrovascular disease evident in CKD.

The limitations of this study are that it is observational in design and therefore, significant associations may not reflect causality. Furthermore, some of the variables including established cardiac disease, stroke, TIA, chronic disease number, and diabetes status are based on participant's self-report of a doctor-delivered diagnosis and could be subject to recall bias. Another limitation of this study is that CKD was defined by eGFR only because there were no urine samples available for albuminuria assessment. The follow-up analysis was limited by sample number because of fewer participants undergoing health assessment with cerebral oxygenation measurement. In addition, although significance testing showed there to be significant differences stemming from the pattern of change in recovery between the eGFR levels, the subgroups are small for these participants in TILDA, and thus subject to large confidence intervals when comparing between levels.

The strengths of this study include the involvement of a large, population-representative cohort of community-dwelling older adults. The comprehensive data collection and standardized health assessments allow for extensive covariate adjustment, further enhancing the robustness of this study. In addition, to the authors' knowledge, this is the first study to examine the dynamic response of cerebral oxygenation continuously after standing, in people with CKD.

In conclusion, this study highlights a significant independent association between CKD and reduced cerebral oxygenation in a large, population-based study. This critical finding suggests that reduced cerebral oxygenation may be a key mechanism underlying the increased rates of cognitive impairment and cerebrovascular disease evident in patients with CKD. Further longitudinal studies in people with CKD are required to deepen our understanding of these findings, to elucidate the implications for cerebrovascular disease and cognitive dysfunction in CKD and to evaluate NIRS as a risk prediction tool for cognitive impairment in CKD. Furthermore, given that this study demonstrates impaired dynamic cerebral oxygenation in early kidney disease, it holds significant implications for the clinical management and care delivery, particularly concerning brain health, for adults with varying degrees of kidney disease.

## Supplementary Material

## Data Availability

Original data generated for the study are or will be made available in a repository subject to controlled access. Data Type: Observational Data; Survey Data. Repository Name: Other. Reason for Restriction: Irish Social Science Data Archive (ISSDA) based in University College Dublin. TILDA offers access to the datasets for research use through pseudonymised publicly accessible dataset files, and through an on-site and remotely accessible Hot Desk Facility. The publicly accessible dataset files are hosted by the ISSDA based in University College Dublin (https://issda.ucd.ie/dataverse/tilda). Researchers can apply for the public datasets directly through ISSDA by completing a data access application and data use agreement available on their website. On approval, ISSDA provides the datasets requested through a secure download link. Further details on accessing the data can find more information and application forms on https://tilda.tcd.ie/data/accessing-data.

## Declarative Statements

This study includes clinical experimentation and received Institutional Review Board or Ethics Committee approval. All patients provided written informed consent. This study includes clinical experimentation and complies with the Declaration of Helsinki.

## Supplemental Material

Supplemental Figure 1. Orthostatic drop in TSI stratified by CKD status.

Supplemental Figure 2. Orthostatic drop in TSI by eGFR Level.

Supplemental Figure 3. Wave six orthostatic TSI by eGFR Level.

Supplemental Figure 4. Wave six orthostatic drop in orthostatic TSI by eGFR level.

Supplemental Table 1. Mixed-effects linear regression models or eGFR level with TSI at wave three as the dependent variable.

Supplemental Table 2. Mixed-effects linear regression models for eGFR level with TSI at wave six as the dependent variable.
