# Supplementary material for: The Association between Cerebral Oxygenation and CKD in Older Adults
Source: Kidney360. 2026 Jan 15;7(5):1046–55. doi: 10.34067/KID.0000001117 (PMC13229429; doi:10.34067/KID.0000001117)
Supplement: Supplementary file 2 [file kidney360-7-1046-s002.pdf]

Supplemental Material

Contents

Supplemental Material 1
Supplemental Figure 1 2
Supplemental Figure 2 3
Supplemental Figure 3 4
Supplemental Figure 4 5
Supplemental Table 1 6
Supplemental Table 2 9
Supplemental Figure Legends 11

Supplemental Figure 1

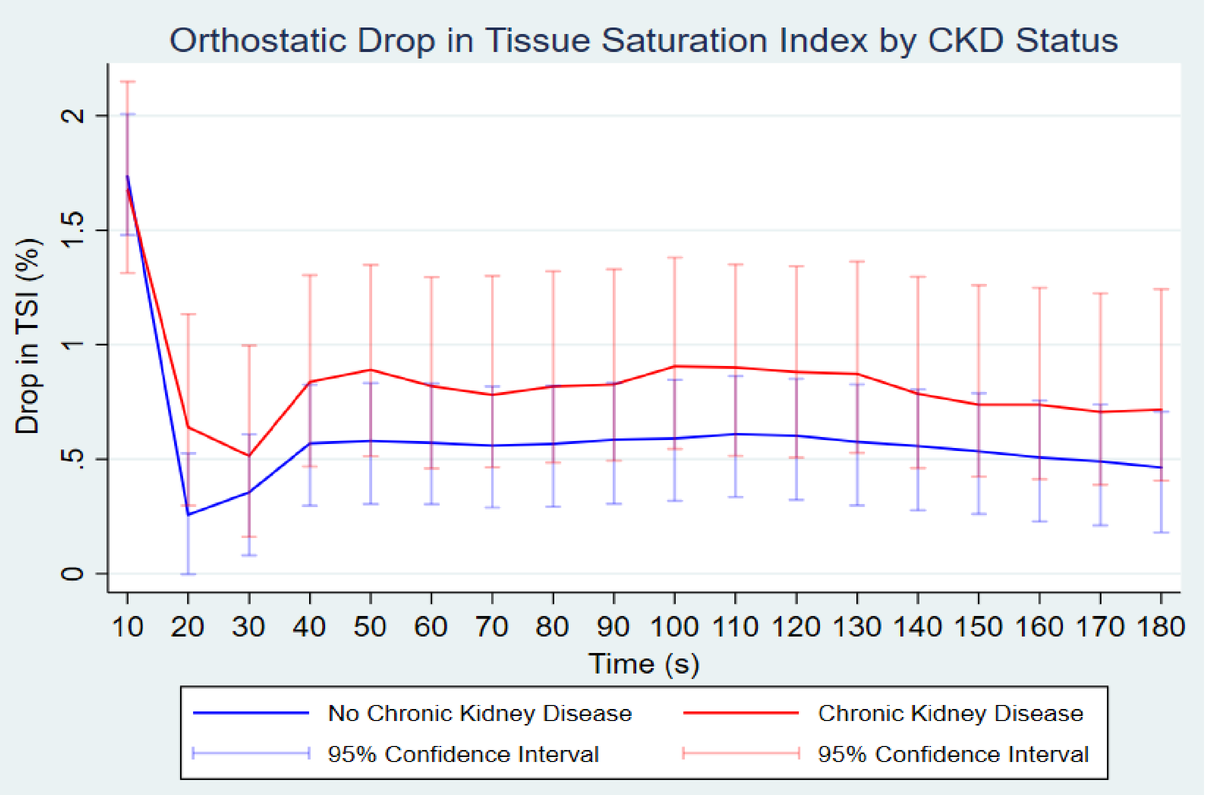

Supplemental Figure 2

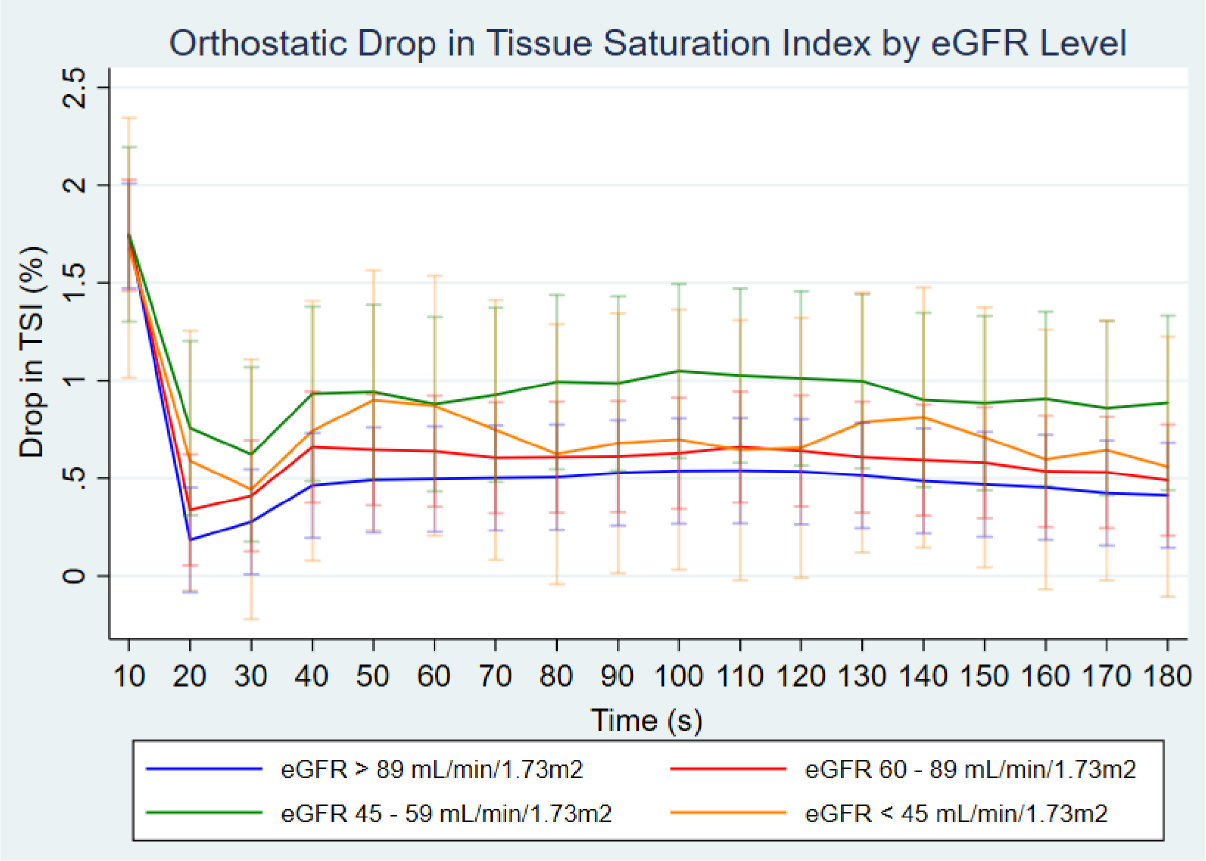

Supplemental Figure 3

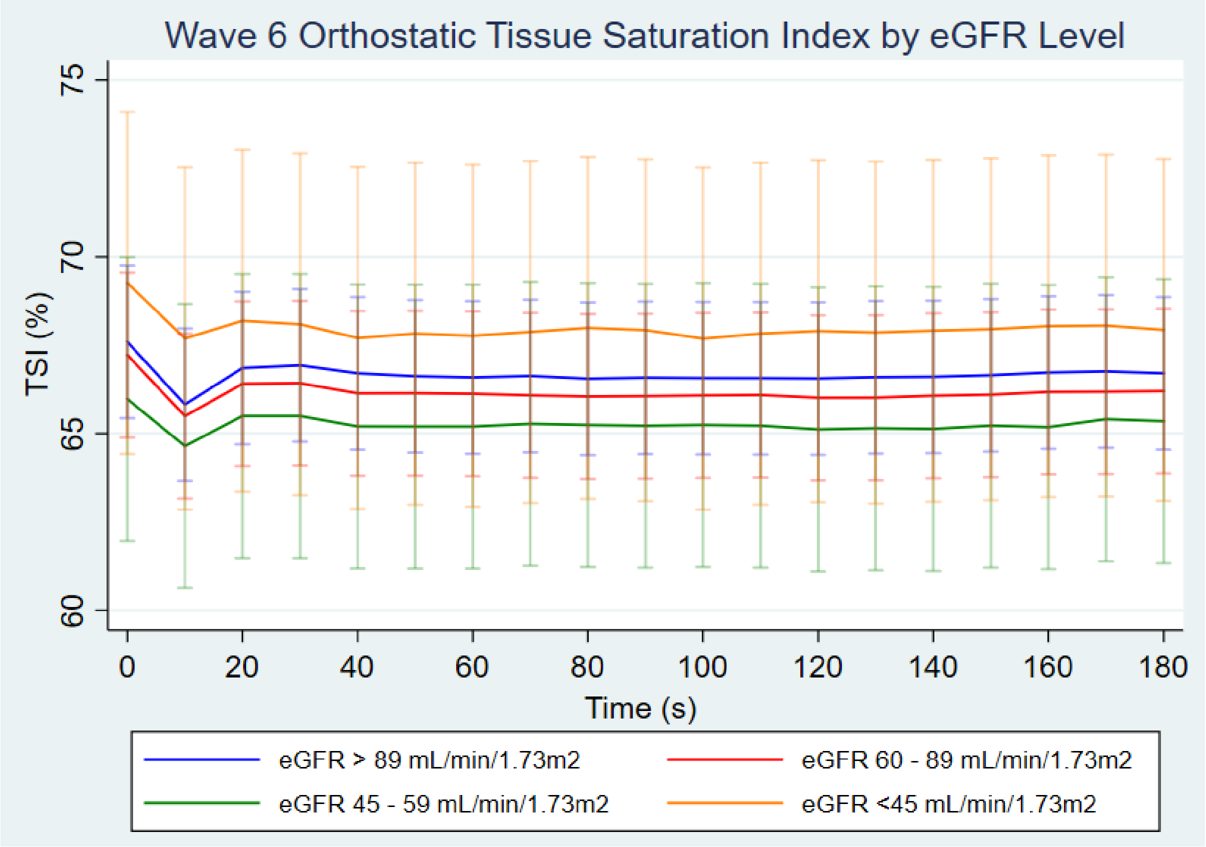

Supplemental Figure 4

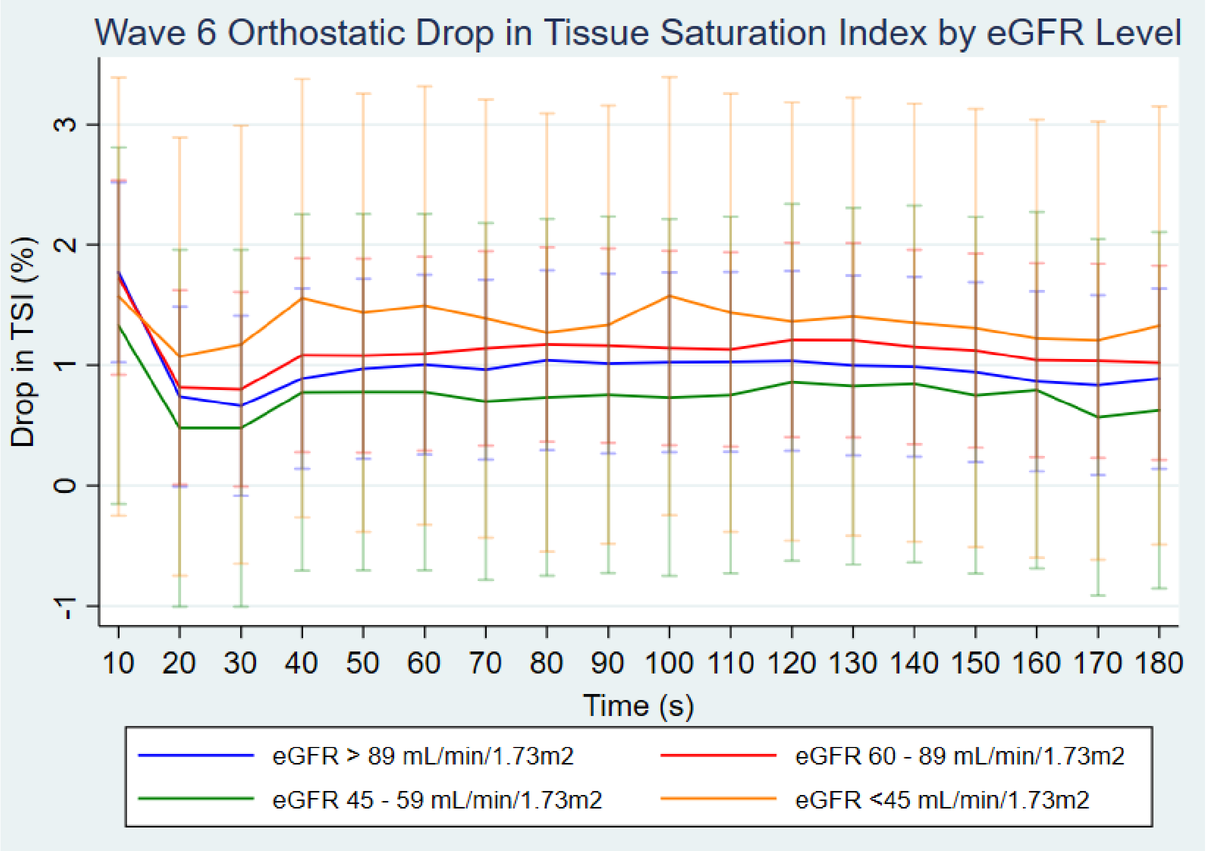

Supplemental Table 1

Supplemental Table 1: Mixed-Effects Linear Regression models for eGFR level with tissue saturation index at wave three as the dependent variable

Wave 3 TSI (Model 3 – time × eGFR Level)	Coefficient (95% CI)	S.E.	Z Score	P Value
10 s eGFR Level 2	-0.00 (-0.11, 0.10)	0.05	-0.05	0.96
10 s eGFR Level 3	-0.01 (-0.24, 0.23)	0.12	-0.05	0.96
10 s eGFR Level 4	0.06 (-0.33, 0.46)	0.20	0.31	0.76
20 s eGFR Level 2	-0.15 (-0.26, -0.05)	0.05	-2.85	0.004
20 s eGFR Level 3	-0.57 (-0.81, 0.34)	0.12	-4.83	0.000
20 s eGFR Level 4	-0.40 (-0.80, -0.01)	0.20	-2.01	0.05
30 s eGFR Level 2	-0.13 (-0.24, -0.03)	0.05	-2.46	0.01
30 s eGFR Level 3	-0.34 (-0.58, -0.11)	0.12	-2.90	0.004
30 s eGFR Level 4	-0.17 (-0.56, 0.23)	0.20	-0.82	0.41
40 s eGFR Level 2	-0.20 (-0.30, -0.09)	0.05	-3.64	< 0.001
40 s eGFR Level 3	-0.47 (-0.70, -0.24)	0.12	-3.95	< 0.001
40 s eGFR Level 4	-0.28 (-0.67, 0.16)	0.20	-1.39	0.17
50 s eGFR Level 2	-0.15 (-0.26, -0.05)	0.05	-2.86	0.004
50 s eGFR Level 3	-0.45 (-0.68, -0.22)	0.12	-3.79	< 0.001
50 s eGFR Level 4	-0.41 (-0.80, -0.01)	0.20	-2.03	0.04
60 s eGFR Level 2	-0.14 (-0.25, -0.04)	0.05	-2.63	0.009
60 s eGFR Level 3	-0.38 (-0.62, -0.15)	0.12	-3.23	< 0.001
60 s eGFR Level 4	-0.37 (-0.77, 0.02)	0.20	-1.86	0.06
70 s eGFR Level 2	-0.10 (-0.21, -0.00)	0.05	-1.92	0.06
70 s eGFR Level 3	-0.42 (-0.66, -0.19)	0.12	-3.58	0.00
70 s eGFR Level 4	-0.25 (-0.64, 0.15)	0.20	-1.22	0.22
80 s eGFR Level 2	-0.10 (-0.21, -0.00)	0.05	-1.90	0.06
80 s eGFR Level 3	-0.49 (-0.72, -0.26)	0.12	-4.11	< 0.001
80 s eGFR Level 4	-0.12 (-0.51, 0.28)	0.20	-0.59	0.555
90 s eGFR Level 2	-0.08 (-0.19, -0.02)	0.05	-1.55	0.121
90 s eGFR Level 3	-0.46 (-0.69, -0.23)	0.12	-3.86	< 0.001
90 s eGFR Level 4	-0.15 (-0.55, 0.24)	0.20	-0.75	0.45
100 s eGFR Level 2	-0.09 (-0.20, 0.02)	0.05	-1.68	0.09

100 s eGFR Level 3	-0.51 (-0.74, -0.28)	0.12	-4.00	< 0.001
100 s eGFR Level 4	-0.16 (-0.56, 0.23)	0.20	-0.80	0.42
110 s eGFR Level 2	-0.12 (-0.23, -0.02)	0.05	-2.24	0.03
110 s eGFR Level 3	-0.49 (-0.72, -0.25)	0.12	-4.10	< 0.001
110 s eGFR Level 4	-0.11 (-0.50, 0.29)	0.20	-0.53	0.60
120 s eGFR Level 2	-0.11 (-0.21, -0.00)	0.05	-1.96	0.05
120 s eGFR Level 3	-0.48 (-0.71, -0.24)	0.12	-4.02	< 0.001
120 s eGFR Level 4	-0.12 (-0.52, 0.27)	0.20	-0.61	0.55
130 s eGFR Level 2	-0.09 (-0.20, 0.01)	0.05	-1.74	0.08
130 s eGFR Level 3	-0.48 (-0.72, -0.25)	0.12	-4.07	< 0.001
130 s eGFR Level 4	-0.27 (-0.67, 0.12)	0.20	-1.35	0.18
140 s eGFR Level 2	-0.11 (-0.21, -0.00)	0.05	-1.99	0.05
140 s eGFR Level 3	-0.41 (-0.65, -0.18)	0.12	-3.50	< 0.001
140 s eGFR Level 4	-0.33 (-0.72, 0.07)	0.20	-1.61	0.11
150 s eGFR Level 2	-0.11 (-0.22, -0.00)	0.05	-2.07	0.04
150 s eGFR Level 3	-0.42 (-0.65, -0.18)	0.12	-3.51	< 0.001
150 s eGFR Level 4	-0.24 (-0.65, 0.15)	0.20	-1.19	0.23
160 s eGFR Level 2	-0.08 (-0.19, 0.02)	0.05	-1.52	0.13
160 s eGFR Level 3	-0.45 (-0.69, -0.22)	0.12	-3.82	< 0.001
160 s eGFR Level 4	-0.14 (-0.54, 0.25)	0.20	-0.71	0.48
170 s eGFR Level 2	-0.11 (-0.21, -0.00)	0.05	-1.97	0.05
170 s eGFR Level 3	-0.43 (-0.67, -0.20)	0.12	-3.66	< 0.001

170 s eGFR Level 4	-0.22 (-0.61, 0.18)	0.20	-1.09	0.28
180 s eGFR Level 2	-0.08 (-0.18, -0.03)	0.05	-1.42	0.16
180 s eGFR Level 3	-0.47 (-0.71, -0.24)	0.12	-3.99	< 0.001
180 s eGFR Level 4	-0.15 (-0.54, 0.25)	0.20	-0.73	0.47

Model 3: adjusted for age, sex, weight, height, education, diabetes, hypertension, cardiac disease, taking anti-hypertensive or anti- depressant medication, depression, stroke history, cognitive impairment, smoking and alcohol history, standing speed and timed-up-and-go.
eGFR Level 1 = eGFR > 89 mL/min/1.73m2
eGFR Level 2 = eGFR 60 – 89 mL/min/1.73m2
eGFR Level 3 = eGFR 45 – 59 mL/min/1.73m2
eGFR level 4 = eGFR < 45 mL/min/1.73m2

Supplemental Table 2

Supplemental Table 2: Mixed-Effects Linear Regression models for eGFR level with tissue saturation index at wave six as the dependent variable

Wave 6 TSI (Model 3 – time × eGFR Level)	Coefficient (95% CI)	S.E.	Z Score	P Value
10 s eGFR Level 2	0.04 (-0.19, 0.27)	0.12	0.36	0.719
10 s eGFR Level 3	0.44 (-0.25, 1.13)	0.35	1.25	0.212
10 s eGFR Level 4	0.20 (-0.60, 1.09)	0.45	0.44	0.659
20 s eGFR Level 2	-0.08 (-0.31, 0.16)	0.12	-0.64	0.520
20 s eGFR Level 3	0.26 (-0.43, 0.96)	0.35	0.74	0.459
20 s eGFR Level 4	-0.3320 (-1.22, 0.56)	0.45	-0.73	0.464
30 s eGFR Level 2	-0.14 (-0.37, 0.09)	0.12	-1.16	0.246
30 s eGFR Level 3	0.19 (-0.51, 0.88)	0.35	0.52	0.600
30 s eGFR Level 4	-0.51 (-1.40, 0.38)	0.45	-1.12	0.262
40 s eGFR Level 2	-0.19 (-0.43, 0.04)	0.12	-1.65	0.100
40 s eGFR Level 3	0.11 (-0.58, 0.81)	0.35	0.32	0.746
40 s eGFR Level 4	-0.67 (-1.56, 0.22)	0.45	-1.47	0.141
50 s eGFR Level 2	-0.11 (-0.34, 0.12)	0.12	-0.92	0.356
50 s eGFR Level 3	0.19 (-0.50, 0.89)	0.35	0.55	0.585
50 s eGFR Level 4	-0.47 (-1.35, 0.42)	0.45	-1.03	0.304
60 s eGFR Level 2	-0.09 (-0.32, 0.14)	0.12	-0.77	0.440
60 s eGFR Level 3	0.23 (-0.47, 0.92)	0.35	0.64	0.522
60 s eGFR Level 4	-0.49 (-1.38, 0.40)	0.45	-1.08	0.279
70 s eGFR Level 2	-0.18 (-0.41, 0.05)	0.12	-1.50	0.133
70 s eGFR Level 3	0.2648 (-0.43, 0.96)	0.35	0.75	0.455
70 s eGFR Level 4	-0.4249 (-1.31, 0.46)	0.45	-0.94	0.349
80 s eGFR Level 2	-0.13 (-0.36, 0.10)	0.12	-1.12	0.263
80 s eGFR Level 3	0.31 (-0.39, 1.00)	0.35	0.87	0.385
80 s eGFR Level 4	-0.23 (-1.12, 0.66)	0.45	-0.51	0.610
90 s eGFR Level 2	-0.15 (-0.38, 0.08)	0.12	-1.27	0.203
90 s eGFR Level 3	0.26 (-0.44, 0.95)	0.35	0.73	0.466
90 s eGFR Level 4	-0.32 (-1.21, 0.57)	0.45	-0.71	0.477
100 s eGFR Level 2	-0.12 (-0.35, 0.11)	0.12	-1.01	0.312
100 s eGFR Level 3	0.29 (-0.40, 0.99)	0.35	0.82	0.410

100 s eGFR Level 4	-0.55 (-1.44, 0.34)	0.45	-1.22	0.224
110 s eGFR Level 2	-0.10 (-0.34, 0.13)	0.12	-0.89	0.375
110 s eGFR Level 3	0.27 (-0.42, 0.97)	0.35	0.77	0.439
110 s eGFR Level 4	-0.41 (-1.30, 0.48)	0.45	-0.90	0.366
120 s eGFR Level 2	-0.17 (-0.41, 0.06)	0.12	-1.48	0.140
120 s eGFR Level 3	0.18 (-0.52, 0.87)	0.35	0.50	0.618
120 s eGFR Level 4	-0.33 (-1.22, 0.56)	0.45	-0.72	0.469
130 s eGFR Level 2	-0.21 (-0.44, 0.02)	0.12	-1.77	0.076
130 s eGFR Level 3	0.17 (-0.52, 0.87)	0.35	0.48	0.629
130 s eGFR Level 4	-0.41 (-1.30, 0.48)	0.45	-0.90	0.369
140 s eGFR Level 2	-0.16 (-0.40, 0.07)	0.12	-1.39	0.163
140 s eGFR Level 3	0.14 (-0.55, 0.84)	0.35	0.40	0.687
140 s eGFR Level 4	-0.37 (-1.25, 0.52)	0.45	-0.81	0.420
150 s eGFR Level 2	-0.18 (-0.41, 0.05)	0.12	-1.51	0.130
150 s eGFR Level 3	0.19 (-0.50, 0.89)	0.35	0.54	0.586
150 s eGFR Level 4	-0.37 (-1.25, 0.52)	0.45	-0.81	0.420
160 s eGFR Level 2	-0.18 (-0.41, 0.05)	0.12	-1.50	0.133
160 s eGFR Level 3	0.07 (-0.62, 0.77)	0.35	0.21	0.837
160 s eGFR Level 4	-0.36 (-1.24, 0.53)	0.45	-0.79	0.432
170 s eGFR Level 2	-0.20 (-0.43, 0.03)	0.12	-1.71	0.087
170 s eGFR Level 3	0.27 (-0.43, 0.96)	0.35	0.75	0.453
170 s eGFR Level 4	-0.37 (-1.26, 0.52)	0.45	-0.82	0.412
180 s eGFR Level 2	-0.13 (-0.36, 0.10)	0.12	-1.11	0.265
180 s eGFR Level 3	0.26 (-0.43, 0.96)	0.35	0.74	0.458
180 s eGFR Level 4	-0.44 (-1.33, 0.45)	0.45	-0.97	0.331

Model 3: adjusted for age, sex, weight, height, education, diabetes, hypertension, cardiac disease, taking anti-hypertensive or anti- depressant medication, depression, stroke history, cognitive impairment, smoking and alcohol history, standing speed and timed-up-and-go.
eGFR Level 1 = eGFR > 89 mL/min/1.73m2
eGFR Level 2 = eGFR 60 – 89 mL/min/1.73m2
eGFR Level 3 = eGFR 45 – 59 mL/min/1.73m2
eGFR level 4 = eGFR < 45 mL/min/1.73m2

Supplemental Figure Legends

Supplemental Figure 1: Orthostatic Drop in Tissue Saturation Index stratified by Chronic Kidney Disease Status.

Supplemental Figure 1 shows the drop in Tissue Saturation Index (TSI) from baseline (0 seconds) at each timepoint after standing on the y-axis with time after standing on the x-axis. Participants with Chronic Kidney Disease (CKD) are represented by the red line while those without CKD are represented by the blue line. The lines represent the predicted values from multilevel modelling with TSI drop as the dependent variable nested within the participant. 95% confidence intervals are represented by capped lines. Models were fully adjusted as per Model 3 in Table 2. TSI was measured by near-infrared spectroscopy and CKD status was based on a 2021 CKD-EPI eGFR of less than 60 mL/min/1,73m^2 using a combination of creatinine and Cystatin C measurements.

Supplemental Figure 2: Orthostatic Drop in Tissue Saturation Index by eGFR Level.

Supplemental Figure 2 shows the drop in Tissue Saturation Index (TSI) from baseline (0 seconds) at each timepoint after standing on the y-axis with time after standing on the x-axis. Participants are stratified by eGFR which was measured using the 2021 CKD-EPI formula using a combination of creatinine and Cystatin C measurements. The blue line represents those with eGFR > 89 mL/min/1,73m^2, the red line represents those with eGFR 60 - 89 mL/min/1,73m^2, the green line represents those with eGFR 45-59 mL/min/1,73m^2 and the yellow line represents those with eGFR < 45 mL/min/1,73m^2. The represent the predicted values from multilevel modelling with TSI drop as the dependent variable nested within the participant. 95% confidence intervals are represented by capped dashed lines. Models were fully adjusted and TSI was measured by near-infrared spectroscopy.

Supplemental Figure 3: Wave Six Orthostatic Tissue Saturation Index by eGFR Level

Supplemental Figure 3 shows the Tissue Saturation Index (TSI) measured at wave six on the y-axis with time after standing on the x-axis. Participants are stratified by eGFR which was measured at

wave three using the 2021 CKD-EPI formula using a combination of creatinine and Cystatin C measurements. The blue line represents those with eGFR > 89 mL/min/1,73m^2, the red line represents those with eGFR 60 - 89 mL/min/1,73m^2, the green line represents those with eGFR 45-59 mL/min/1,73m^2 and the yellow line represents those with eGFR < 45 mL/min/1,73m^2. The lines represent the predicted TSI values derived from multilevel modelling with TSI as the dependent variable nested within the participant. 95% confidence intervals are represented by capped lines. Models were fully adjusted as per Model 3 in Supplementary Table 1. TSI was measured by near-infrared spectroscopy.

Supplemental Figure 4: Wave Six Orthostatic Drop in Orthostatic Tissue Saturation Index by eGFR Level

Supplemental Figure 4 shows the drop in Tissue Saturation Index (TSI) from baseline (0 seconds) at each timepoint after standing at wave six on the y-axis with time after standing on the x-axis. Participants are stratified by eGFR which was measured at wave three using the 2021 CKD-EPI formula using a combination of creatinine and Cystatin C measurements. The blue line represents those with eGFR > 89 mL/min/1,73m^2, the red line represents those with eGFR 60 - 89 mL/min/1,73m^2, the green line represents those with eGFR 45-59 mL/min/1,73m^2 and the yellow line represents those with eGFR < 45 mL/min/1,73m^2. The lines represent the predicted values from multilevel modelling with TSI as the dependent variable nested within the participant. 95% confidence intervals are represented by capped dashed lines. Models were fully adjusted and TSI was measured by near-infrared spectroscopy.
